# Measuring anxiety and fear of Covid-19 among older people: psychometric properties of anxiety and fear of Covid-19 scale (AMICO) in Spain

**DOI:** 10.1186/s12889-022-13960-w

**Published:** 2022-08-20

**Authors:** Aurora Vélez-Morón, Rafael T. Andújar-Barroso, Regina Allande-Cussó, Juan Jesús García-Iglesias, Gregoria Aquino-Cárdenas, Juan Gómez-Salgado

**Affiliations:** 1 Personality, Psychological Assessment and Treatment Area, Department of Clinical and Experimental Psychology, Faculty of Education, Psychology and Sports Sciences, University of Huelva, 21071 Huelva, Spain; 2 Department of Nursing, University of Seville, Spain. Avenzoar st, 6, 41009 Sevilla, Spain; 3 Department of Sociology, Social Work and Public Health, Faculty of Labour Sciences, University of Huelva, 21007 Huelva, Spain; 4 Psychobiology Area, Department of Clinical and Experimental Psychology, Faculty of Education, Psychology and Sports Sciences, University of Huelva, 21071 Huelva, Spain; 5 Safety and Health Postgraduate Programme, Universidad Espíritu Santo, Guayaquil, 092301 Ecuador

**Keywords:** Anxiety, Fear, COVID-19, Elderly people, Mental Health, Assessment

## Abstract

**Background:**

The elderly population has proven to be a particularly vulnerable group with regard to the effects of the COVID-19 pandemic. The aim of this study was to study the psychometric properties of the Ansiedad y MIedo a Covid-19 scale (AMICO) on a population-based sample of elderly people.

**Methods:**

A descriptive and psychometric cross-sectional study, based on questionnaires, was carried out. An exploratory and confirmatory factor analysis was performed, as well as a bivariate analysis between the different sociodemographic variables with respect to the total scale score.

**Results:**

A sample of 720 adults over 65 years of age was obtained, 52.2% of whom were women. The structure of the factor of the scale showed two factors (fear and anxiety) and was confirmed with good fit parameters. The overall reliability of the scale in terms of internal consistency was α = 0.94.

**Conclusions:**

The AMICO scale is a valid and reliable instrument to measure anxiety and fear of COVID-19 in the Spanish population over 65 years of age. Women and subjects with a partner showed the highest values of fear and anxiety.

## Introduction

The psychological consequences of the COVID-19 pandemic for the general population are being reported in multiple studies, which find higher levels of anxiety, fear and stress in almost all sectors of the population. Furthermore, levels of fear could be an indicator of the risk that subjects assume in the face of the possibility of contagion. They could also indicate the tendency to comply with measures (aimed at avoiding contagion), as data from some studies suggest, as a way to explain the greater effect on certain sectors of the population, as is the case of the elderly [1].

Previous studies on disaster situations have highlighted the greater vulnerability of the elderly in these scenarios, recognising this group as one that is not adequately cared for in these cases, which is why care should be prioritised to reduce their morbidity and mortality [2]. This population has proven to be a group particularly at risk as regards the effects of the COVID-19 pandemic [1, 3, 4], suffering higher rates of hospital admissions, more sequelae following illness, and higher mortality than other population age groups, both for institutionalised and non-institutionalised elderly [5–7].

In this context, it has been found that the mental health risk of the population has increased [8, 9], and it has even been suggested that it could be higher for the older age group as we consider that the psychological effects could extend to the medium and long term [10–13]. Despite this, as several studies show, the incidence of psychological distress found in older age groups tends to be at rates not significantly higher than in other age groups of the population [4, 14] and even at lower levels than younger people, i.e. below the age of 60 [14, 15]. Thus, older age has been considered a protective factor against adverse psychological effects of the pandemic [14, 16–19].

In the search for useful instruments for the assessment of emotional and psychological states related to the pandemic, tools such as the Coronavirus Anxiety Scale, designed by Lee, have been developed. This 5-item scale measures the COVID-19 anxiety construct, and obtained a Cronbach's alpha value of 0.93 and optimal model fit values by confirmatory factor analysis [20]. Another scale, the FCV-19 (Fear of COVID-19) designed by Ahorsu et al., has been created [21]. This one was developed for the specific measurement of fear of COVID-19 as a psychological problem derived from the pandemic, and it was validated in a sample of Iranian population over 18 years of age (*n* = 717; mean age = 31.25 ± 12.68), showing adequate validity and reliability, with a unidimensional structure composed of seven items and no significant differences in terms of sex and age. Validation studies of the FCV-19 have been carried out in the context of a wide variety of cultures and countries in Europe, America and Asia [22–31]. All of them note the robustness in the measurement of fear of COVID-19, the reliability and validity of the instrument, its unidimensional structure, as well as the fact that practically all studies focus on samples of the general population and very scarcely on the elderly.

Among the few studies that have carried out FCV-19 assessments specifically in the elderly population are the study by Li et al. [32], with a Taiwanese sample (*n* = 139; mean age: 71.73), the study by Pakpour et al. [22], comparing the latter sample with an Iranian population (*n* = 144; mean age: 65.59), or that by Mistry et al. [23] with a Bangladeshi sample (*n* = 1032,; age ≥ 60). These authors reported respective mean FCV-19 values of 1.8 out of 5 (Taiwan), 3.36 out of 5 (Iran), and 2.8 out of 5 (Bangladesh), all similarly low levels of fear of COVID-19 as compared to mean scores of 4 out of 5 reported by the original general population study of the scale [21].

In order to measure not only the fear construct, or anxiety construct, separately, based on the work of Ahorsu et al. [21], the AMICO scale (Escala de Evaluación de la Ansiedad y MIedo a la COvid-19 – Anxiety and Fear of Covid-19 Assessment Scale) was designed [33]. The scale has proven to be valid and reliable as a screening instrument for a Spanish general population sample [34].

Work with the AMICO scale has so far been carried out in the general population in Spain, and the scale shows stability and absence of differences in terms of age [34, 35].

In any case, there is still a lack of studies focusing on older people, even more so due to the pandemic situation, which makes a specific approach to this population group necessary [36]. Thus, the main objective of this paper is to study the specific psychometric properties of the AMICO scale in a population-based subsample of older people, as well as to test its factor structure and determine its validity and reliability as a measure of anxiety and fear of COVID-19 in this age group.

## Method

### Design

Descriptive and psychometric cross-sectional study, based on questionnaires.

### Participants

The population of people over 65 years of age in Spain amounts to 9,000,000 people [37]. The calculation of the required sample size was 270 subjects, considering a confidence level of 95%, a heterogeneity of 50%, a 25% loss rate, and a margin of error of 5%. Nevertheless, data were eventually collected from 720 subjects.

Convenience sampling was carried out, and the sample was accessed with the help of the coordinators of the Aulas de la Experiencia (Schools of Experience, a Spanish service provided for the elderly who wish to attend university-like studies) of all the universities in the country. A presentation of the study was distributed by email, together with the request for informed consent and the link to the Google Forms© questionnaire to all people over 65 years of age enrolled in one of the university training plans for the elderly. Likewise, once the subject accessed the questionnaire via the corresponding link, questions were asked about the legal conditions and the consent to be able to access the survey.

### Variables

The online questionnaire contained socio-demographic variables (sex, age, province of residence, marital status, employment status, level of education, questions related to COVID-19 contacts and infections, and self-perceived level of health). In addition, among other measures, a scale variable was included for the measurement of anxiety and fear of COVID-19.

### Instrument

For the assessment of the presence of anxiety and fear of COVID-19, the Anxiety and Fear of COVID-19 (AMICO) scale was used, designed and validated in previous studies, with a 2-factor dimensional structure and 16 items that explained 64.8% of the variance [33, 34]. The reliability study offered a value of α Cronbach = 0.92 [33, 34]. The response options of the AMICO scale ranged from 1 to 10 points, where 1 indicates strongly disagree, and 10 indicates strongly agree. The cut-off point for the general population was set at 6.4 points, above which anxiety and fear of Covid-19 is considered to exist [34].

To study the convergent validity of the scale, and to provide data on its criterion validity, the General Health Questionnaire (GHQ-12), which measures the presence of emotional distress —closely related to the presence of anxiety and fear—, was included in the study [38]. It was developed by Goldberg, and it has been translated and validated into many languages, with Cronbach's alpha values ranging from 0.82 to 0.86 [39]. In its Spanish version, the scale obtained Cronbach's alpha values ranging from 0.86 to 0.76 [40].

### Data analysis

Univariate and bivariate statistical analyses were performed using SPSS Statistics v.26 [41]. For the psychometric study, the total sample was randomly divided into two sub-samples; thus, on sub-sample 1, an exploratory factor analysis was performed to determine the factor structure of the scale and the percentage of variance explained. The Kaiser method was used to identify the relevant number of factors, and the principal component extraction and varimax rotation were used to obtain the factor loadings. Items with loadings below 0.5 were eliminated. This was followed by a confirmatory factor analysis, also using AMOS© software [42], on sub-sample 2. To assess the goodness-of-fit of the confirmatory models, the following indexes were used: the penalty function (Chi-squared over degrees of freedom (CMIN / DF) (values ≤ 3 indicated a good fit); the RMSEA (Root Mean Square Error of Approximation) index (values ≤ 0.08 indicated a good fit); NFI (Normalised Fit Index), CFI (Comparative Fit Index); and TLI (Tucker-Lewis Index) (values ≥ 0.95 indicated a good fit) [43]. After this, to study the unidimensionality of the scale, a two-factor model was analysed, considering the same first-order factors validated by the recently performed CFA and also a second-order factor (bifactor) in which each item was also subsumed [44]. The Dueber Bifactor index calculator [44]. was used to calculate the goodness-of-fit parameters of the bifactor model. Specifically, the percent of uncontaminated correlations (PUC) was used, which represents the percentage of variance that corresponds only to the overall dimension, the percentage of explained common variance (ECV) which is the proportion of total variance that is explained by each factor (general and specific)—for specific factors the ECV reflects the strength of a specific factor in explaining the variance of the items that load on it and the Omega Hierarchical (OmegaH), which reflects the percentage of systematic variance of the total score that can be attributed to individual differences in the general factor. Regarding the cut-off points for these indices, Reise et al. suggest that PUC values > 80, together with LCS values > 60 and Omega H > 80, would indicate that the presence of multidimensionality would not be too severe to rule out unidimensionality of the scale [45].

Regarding criterion validity, this could not be studied by comparison with a gold standard, but the mean score and its distribution in quartiles were used for the proposed levels of anxiety and fear in the population of people over 65 years of age. The existence of statistically significant differences between levels was tested by the U-Mann Whitney test.

In addition, to study the reliability of the scale, Cronbach's alpha coefficient was calculated for both the factor solution obtained by exploratory analysis and the one obtained by confirmatory analysis. Likewise, based on new recommendations for the study regarding the reliability of measurement scales, the McDonald’s omega coefficient was calculated, which confirms the premise of Tau-equivalence and is a more robust indicator of the reliability of the scale [46]. In addition, the McDonald's omega coefficient was corrected to control for the possible effect of overestimation on the reliability indices [47]. In the same sense, the reliability of the proposed cut-off points was studied and the Livingston coefficient was calculated, whose premise is the study of reliability based on the deviation from the cut-off point [48, 49].

For the bivariate study, the normality of the data distribution was analysed using the Kolmogorov–Smirnov test, and a significance of 0.04 was obtained, showing non-normality. Contrast tests such as Mann–Whitney U and Kruskal–Wallis were therefore used. Kendall's Tau-b statistic was used to study the correlation between quantitative variables. The correlation between the AMICO scale total score and the GHQ scale total score was also calculated using Kendall's Tau-b statistic.

### Ethical aspects

This study is part of the IMPACTCOVID-19 project, which aims to assess the impact of the Covid-19 pandemic on the emotional well-being and psychological adjustment of the general population in Spain, which was granted permission to be implemented by the Ethics and Research Committee of the Regional Government of Andalusia (Ref. PI 036/20). The study also complies with the guidelines of the Declaration of Helsinki of Ethical Principles for Human Research [50, 51] and the state regulations on biomedical research [52].

All subjects in the sample confirmed their voluntary and confidential participation in the study by means of a specific box, in which they had to tick the option "I agree to participate". Otherwise, the application did not allow access to the questionnaire.

## Results

### Descriptive analysis

The total sample consisted of 720 subjects, over 65 years of age and resident in Spain. Of this sample, 52.2% were women with a mean age of 69.4 years (SD = 3.8). Likewise, 63.8% were married, 14.4% divorced, 12.1% widowed, and 9.7% single. In addition, 97.9% of the sample were retired, 1.1% had never worked, and 0.9% were still working. With regard to educational level, 68.1% had higher education, 24.9% had vocational training, and the remaining 7% had primary and secondary education (see Table 2).

Regarding the Covid-19 diagnosis variable, only 6% of the sample had been infected with Covid-19 at the time of data collection, and 15.6% had required isolation due to close contact with a positive case. In addition, the self-perceived level of health, on a range of 0 to 10 points, scored a mean of 7.56 points (SD = 1.3) (see Table 1).

**Table 1:** Sample description and hypothesis testing

	**Total sample** **(*n* = 720)**	**Mean score**	Contrast hypothesis*
**Sex**			
Female	376 (52.2%)	5.35	**p = 0.001**^**a**^
Male	344 (47.78%)	4.86	
**Age**			
Mean (SD)	69.4 (3.8%)		Tau = -0.08^b^
**Marital status**			
Married	454 (63.8%)	5.14	**p = 0.049**^**c**^
Divorced	104 (14.4%)	4.76	
Widow/er	87 (12.1%)	5.34	
Single	75 (9.7%)	5.37	
**Work situation**			
Retired	705 (97.9%)	5.11	p = 0.55^c^
Working	4 (0.9%)	4.38	
Never worked	11 (1.1%)	5.73	
**Educational level**			
Higher studies	491 (68.1%)	5.21	p = 0.23^c^
Vocational training	179 (27.9%)	5.37	
Primary and/or Secondary	50 (7%)	5.49	
**Covid-19** d**iagnosis**			
No	677 (94%)	5.15	**p = 0.029**^**c**^
Yes	43 (6%)	4.54	
**Isolation by contact**			
No	608 (84.4%)	5.15	p = 0.23^c^
Yes	112 (15.6%)	4.92	
**Self-perceived health**			
Mean (SD)	7.56 (1.3)		Tau = -0.14^b^

Values in bold are significant *p*-values

^a^U Mann Whitney

^b^ Tau B de Kendall

^c^H Kruskal–Wallis

^*^Non-parametric contrast statistics

The mean score on the AMICO scale was 5.11 points (SD = 1.83). The bivariate analysis reported no statistically significant differences between the mean scores on the AMICO scale for the different categories of the employment status, academic level, and isolation by contact variables. On the other hand, the contrast statistics did show significant differences for the sex, marital status, and Covid-19 diagnosis variables (see Table 2). Thus, women, single or widowed, and those who had not been infected by SARS-CoV-2 virus presented higher mean scores on the AMICO scale. On the other hand, no association was found between the mean scores of the AMICO questionnaire and the age and self-perceived health variables (see Table 2).

**Table 2:** Exploratory factor analysis

SCALE ITEMS	FACTORS	
	**Anxiety**	**Fear**
ITEM_1		.742
ITEM_2		.680
ITEM_3		.831
ITEM_4		.812
ITEM_5	.763	
ITEM_6	.700	
ITEM_7	.836	
ITEM_8	.872	
ITEM_9	.652	
ITEM_10	.752	
ITEM_11		.750
ITEM_12		.760
ITEM_13		.702
ITEM_14		.736
ITEM_15	.763	
ITEM_16	.764	

### Psychometric analysis of the AMICO scale

#### Study of construct validity and reliability

The total sample of 720 subjects was randomly subdivided into two sub-samples of 360 subjects. On sub-sample 1, the Kaiser-Meier-Olkin statistic obtained a value of 0.93 (*p* = 0.001) and Bartlett's test of sphericity obtained a value of *p* = 0.001. By means of exploratory factor analysis (EFA), the dimensional matrix of the 16 items was extracted, with 2 factors explaining 65.2% of the variance (Table 2). Items 6 and 7 were eliminated as their factor loadings were all < 0.5. The reliability study gave a total value of α Cronbach = 0.94, and of 0.94 for factor 1 (Anxiety) and 0.92 for factor 2 (Fear). The intraclass correlation coefficient was 0.94 (*p* = 0.001).

Initially, a Confirmatory Factor Analysis (CFA) was performed on the basis of a model with two factors (anxiety and fear) which, despite offering an adequate fit, required a high number of correlations between errors. Taking into account this circumstance, a two-factor model (as a second-order CFA) was chosen to test both the structured organisation of the scale and its unidimensional character, and the preferential use of a total score.

**Fig. 1:**
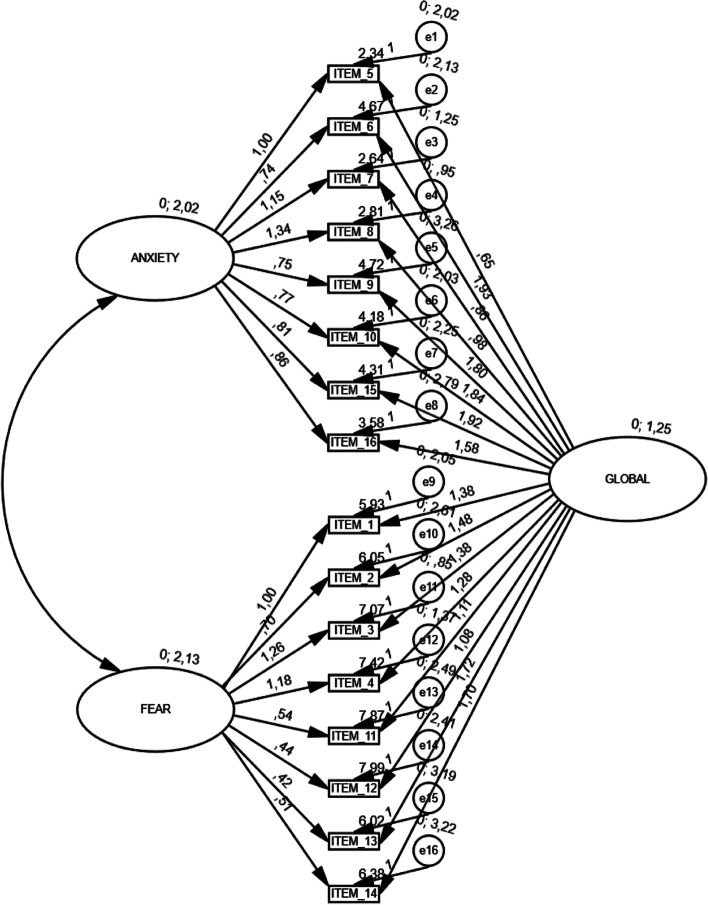
Bifactor Structure Diagram of the scale

Therefore, the confirmatory factor analysis (CFA) was performed to study the validity of the construction, with subsample 2, which yielded the following values: CMIN / DF = 3.79;: NFI = 0.97; TLI = 0.96; CFI = 0.97; RFI = 0.96; and RMSEA = 0.065 (see Fig. 1). The reliability study of the factor solution validated by CFA gave a total value of Cronbach’s α = 0.94, and the McDonald's omega coefficient value for the composite reliability study was 0.91. Furthermore, despite not including correlated residuals in the model, the correction of the omega index was executed, as proposed by Dominguez [47] and Viladrich,et al. [53], and a corrected omega value of 0.90 was obtained.

On the other hand, another structural equation model was also estimated to relate the scale to another variable that assesses the presence of emotional distress used in the study, such as the GHQ-12 scale score. The results of the CFA reported values of CMIN / DF = 12.169; NFI = 0.91; TLI = 0.90; CFI = 0.91; RFI = 0.90; and RMSEA = 0.07. Similarly, the model did not require the incorporation of correlation between residuals of any of the items for better fit.

In relation to the Bifactor model indices, a value of PUC = 0.63, ECV = 0.65, and Omega H = 0.82 was obtained.

#### Proposed stratification of anxiety and fear levels

The study of the correlation between the GHQ total score and the AMICO scale gave a Kendall’s Tau-b value of 0.6 (*p* = 0.001).

The mean score on the AMICO scale was 5.11 points (SD = 1.82), with a range of scores from 1.13 to 9.81. Based on the distribution of mean AMICO scale scores, quartile 1 had a score of 3.75, quartile 2 a score of 5.06, and quartile 3 a score of 6.4. Thus, the following correction scale is proposed for the AMICO scale: low level, from 0 to 5 points; intermediate level, from 5.01 to 6.4 points; high level, a score of more than 6.41 points.

Regarding the reliability of the cut-off points, the first cut-off of 5 points had a Livingston coefficient of 0.94; the second cut-off of 6.4 points had a Livingston coefficient of 0.9; and the third cut-off of 6.41 points had a Livingston coefficient of 0.91.

**Fig. 2:**
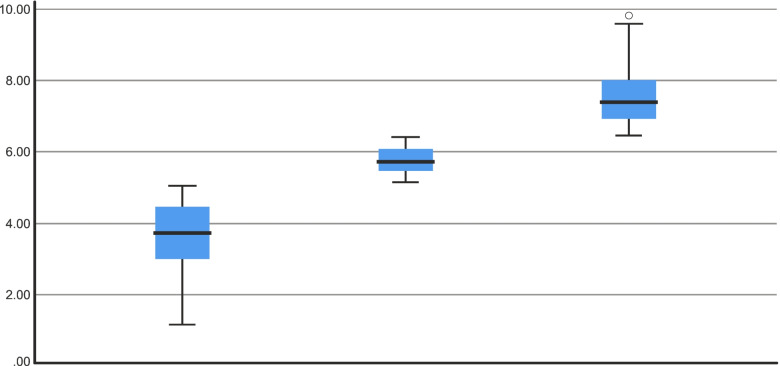
Box plot for each level of the AMICO scale

Figure 2 shows the distribution of the sample in the three levels of anxiety proposed by means of a box plot:

The analysis of the statistical significance of the differences between the different levels identified, using the Mann Whitney U statistic for each pair of levels tested, always offered a value of *p* = 0.001. Thus, there are significant differences between the levels identified and it is therefore possible to consider and identify them as a correction of the AMICO scale for people over 65 years of age.

## Discussion

The results of the exploratory factor analysis (EFA) clearly show a distribution consistent with the previous study by Gómez-Salgado et al. [34] around two factors, which explain a total variance of 66.6%, somewhat higher than in the general population sample. The distribution of the items around the "anxiety" and "fear" factors was identical to that reported by the previous study, which confers consistency and stability, both to the measurement of the overall construct and to its factorial components. In addition, goodness-of-fit values were equally good according to the results of the confirmatory factor analysis (CFA). Similarly, the second calculated structural equation model, considering the correlation between the AMICO scale and the GHQ-12 scale, also reported optimal fit values, which would support the adequate reliability of the AMICO scale, as well as the adequate construct validity of the instrument.

Regarding the cut-off points for the Bifactor model indices, Reise et al. suggest that PUC values > 80, together with LCA values > 60 and Omega H > 80, would indicate that the presence of multidimensionality would not be too severe to rule out the relevance of unidimensionality of the scale [45]. In this sense, the similarity between the found values of total Omega (0.90) and Hierarchical Omega (0.82), following Green and Yang [54], would also point to evidence in favour of the unidimensionality of the instrument. This validated dimensional structure would justify the use of the total scale score as an appropriate measure of COVID-19 fear and anxiety. The instrument is highly reliable, with internal consistency data even somewhat higher (Cronbach's alpha 0.94) than those reported in the general population study [34], both for the total scale and for each of the separate factors ("anxiety" and "fear"). In addition, the present study provides composite reliability data by means of the McDonald's omega coefficient, which are optimal and provide greater robustness to the reliability study of the scale. The results of the corrected Omega H and Omega index suggest that the reliability values remain in a high range; they are similar and close to the alpha value itself, which would indicate, in addition to a low overestimation of reliability, that a "clinical" use of the instrument might be less appropriate (being in the 0.90 range). Furthermore, it seems that a clinical use of the instrument might be less appropriate (because it is within the 0.90 range), but that its use for group estimates is highly reliable (attributable to values somewhat lower than 0.90 [55], which is precisely the aim of the AMICO scale.

On the other hand, the AMICO scale shows moderate but significant convergent validity with the GHQ scale. However, it should be noted that the GHQ scale assesses the presence of psychological distress [39, 40], not only in terms of anxiety and fear, but also in terms of depression, which could justify a moderate convergence between the two scales. Thus, it can be said that the AMICO scale for older people has criterion validity, also supported by the fact that the existing literature reports higher levels of emotional distress in women in Spain [56, 57], and the results of the present study conclude the same findings.

In comparison with measures of fear taken in other studies with the FCV-19 scale, especially those carried out on the elderly population, the internal consistency values are slightly lower than those resulting from the AMICO scale: α = 0.79 in a Taiwanese sample by Li et al. [32]; α = 0.89 for the Bangladeshi sample in the study by Mystry et al. [23] or α = 91 in the Iranian sample in the study by Pakpour et al. [22], something that persists in the various general population studies in samples from various countries [36]. Probably, the inclusion of the new items in the AMICO scale and the higher final number of items (16 items) with respect to the FCV-19 (7 items) could explain the higher internal consistency values, an indication of adequate congruence in the measurement of the construct, also for the elderly population.

The levels of anxiety and fear of the COVID-19 seem to be lower for this age group over 65 (mean = 5.11 ± 1.83) than for the general population, as compared to the data reported by the authors of the AMICO scale in their previous study [34, 35], who found mean values of 5.54 (± 1.83) and a range with higher upper limit values (from 1.22 to 10 points). Other studies with older populations also found lower values of fear in older adults than other lower age groups or compared to the general population [22, 23, 32, 58, 59], which is corroborated by the meta-analysis by Lin et al. [36] with data from the use of the FCV-19 scale in eleven studies from different countries and general population samples of different ages. In this sense, another work with samples of older people [60] reports relatively lower values of fear of COVID-19 than in the general population in the same context, based on FCV-19 scale measures [61], with no differences between age groups above 65 years (65–74, 75–85, and 85–94 years).

Although no statistically significant differences were found with respect to their level of education, it did appear that a higher level of education tended to be associated with a lower level of anxiety and fear of COVID-19, occurring in the same sense, albeit more intensely and with statistical significance, in previous studies with the AMICO scale in the general population [34, 35]. Studies of fear of COVID-19 measured with the FCV-19 scale report results similar to those of our study. Thus, studies such as the one by Mistry et al. [23] or by Pakpour et al. [22], also with samples of elderly people, do not find differences in fear as an effect of the degree of educational background. The data of Gokseven et al. [60], although lacking statistical significance, even point in the opposite direction, so that in the Turkish elderly population, those subjects with higher levels of education would have a higher level of fear of COVID-19. Yağar [58], on the other hand, finds in a sample of older adults also in a Turkish context that the lower level of education was clearly associated with a higher level of fear of COVID-19. However, this author suggests that the key element could be the "health education" factor which, probably favoured by higher levels of education, would have the effect of reducing levels of fear. In the general population with lower mean age, on the other hand, a clearer and more generalised inverse relationship seems to be found between educational level and fear of COVID-19, so that lower levels of educational attainment would be associated with higher values on the FCV-19 scale [62].

Similar to data reported for the general population with the AMICO scale [34, 35], significantly higher values for anxiety and fear on the COVID-19 were found in females than in males. This was to be expected based on the higher levels of anxiety that the female population tends to manifest in a generalised way in almost all social contexts and adult age groups [63, 64]. These results are consistent with those found by Mistry et al. in a sample of older people in Bangladesh, where women scored significantly higher on FCV-19 than men [23]. However, they are contrary to those reported by Li et al. [32] in a Taiwanese sample of older people, where they found a lack of significant differences between sexes, with even lower fear values in females than in males (measured with the FCV-19). However, some studies specifically avoid intersex comparative analyses, either because they are not part of the objectives, as in the case of Pakpour et al. [22] or because of methodological inadequacy due to the small sample size for one of the sexes, as in the case of the study by Soraci et al. [24].

In relation to the marital status, data from this study show higher levels of anxiety and fear of COVID-19 in single and widowed subjects than in married subjects, contrary to what was found in the general population, where the latter showed the highest values on the AMICO scale [34, 35]. Data reported on the use of the FCV-19 scale in some studies also seem to find higher levels of fear of COVID-19 in unmarried subjects [58, 60, 65], although other studies find reverse results [29, 62]. Nevertheless, the results of some works point to the fact that having a partner may be a protective factor against the psychological and psychiatric effects of the pandemic [66] and others that it is the fact of living alone that would confer the greatest propensity to higher levels of fear, especially in the elderly [23, 60].

On the other hand, the results show that not having had a diagnosis of COVID-19 seems to introduce in the elderly population a favouring element of higher levels of anxiety and fear of COVID-19, something that, although lacking statistical significance, was also observed in the data reported through the AMICO scale in the general population [35]. These results are in line with the data provided in the study where the validation of Lee’s Coronavirus Anxiety Scale [20] was presented. However, they are contrary to those found by Lee et al. [67], who reported higher levels of COVID-19 anxiety in those who had a previous diagnosis of the disease, using a much younger sample (mean age 35.91 ± 11.73 years).

Likewise, in line with previous studies on anxiety levels in general (measured with the STAI scale) that also note a trend towards lower values in the older population [64, 68, 69], it would be important to analyse, beyond the differences in levels in terms of anxiety or fear, the particular circumstances, personal conditions and life events that could be found to favour higher levels of anxiety or fear of COVID-19.

The AMICO scale validated in the elderly population could be a useful tool, both for identifying mental health risks arising from emotional consequences of pandemic-related experiences [1, 21], and for improving the planning and implementation of preventive behaviours and attitudes towards COVID-19. It should also be considered as a possible "predictor" of compliance with public health measures, given the role that fear and anxiety appear to play in the performance of hygiene-enhancing and distancing behaviours related to COVID-19 [70]. However, other studies that examine, beyond anxiety or fear level differences, particular circumstances, personal conditions and life events, which might be found to favour higher levels of anxiety or fear of COVID-19, should be considered in the future.

The sample of elderly people used, although representative in number, was collected by non-randomised procedures, presenting a varied distribution in the different Spanish provinces in which responses were sought to be collected, with the presence of subjects being low or non-existent in some cities. Likewise, the sample refers to a group of elderly people which, due to links with the university context (lifelong learning programmes for the elderly in the university context), may not be representative of the social context of elderly people in general. In this respect, new studies that consider other educational levels in the elderly are needed.

On the other hand, in contrast with the validation study of the Coronavirus Anxiety Scale [20], it was not possible to carry out concurrent validity analyses due to the lack of relevant variables that would allow a standard reference of measurement (gold-standard) in the field of anxiety/fear. Thus, despite the fact that the proposed cut-off points showed high reliability, it was not possible to rigorously establish optimal cut-off points by studying the sensitivity and specificity of the instrument with the relevant ROC (Receiver Operating Characteristic) curve, something that will remain pending for subsequent studies.

## Conclusions

The results of the present study show that the AMICO scale is a valid and reliable instrument for measuring anxiety and fear of COVID-19 in the over-65 population, with a robust bifactor structure (anxiety and fear) similar to that found for the general population.

The cut-off points would place fear and anxiety to COVID-19 at lower levels than in the general population, although the clinical significance would probably tend to be at similar values. However, studies of specificity and sensitivity based on gold standards are necessary in this respect.

## Data Availability

The datasets used and/or analysed during the current study are available from the corresponding author on reasonable request.
